# Experiences of caregivers and healthcare providers regarding health services for children with Down syndrome in Karachi; Pakistan

**DOI:** 10.1371/journal.pgph.0006225

**Published:** 2026-04-30

**Authors:** Zeeluf M. Qaisar, Salman Kirmani, Imran Naeem, Muhammad Asim, Laila Akbar Ladak

**Affiliations:** 1 School of Nursing and Midwifery, The Aga Khan University, Karachi, Pakistan; 2 Division of Women and Child Health, The Aga Khan University, Karachi, Pakistan; 3 Department of Community Health Sciences, The Aga Khan University, Karachi, Pakistan; Centre of Biomedical Ethics and Culture, PAKISTAN

## Abstract

This study aimed to investigate the experiences and perceptions of caregivers seeking healthcare services for children with Down Syndrome, and those of healthcare providers offering these services in Karachi, Pakistan. A total of 23 In-depth interviews were conducted with the study participants comprising of 10 caregivers (mothers and fathers) and 13 healthcare providers (paediatricians and therapists). Participants were selected through purposive sampling and interviewed using a semi-structured interview guide at a private NGO and a tertiary care hospital. The collected data underwent deductive content analysis, guided by the socio-ecological framework, to comprehensively explore the various factors. Experiences clustered across socio‑ecological levels. Intrapersonally, caregivers moved from shock and grief to faith‑based acceptance that sustained caregiving. Interpersonally, delayed/missed diagnosis, inadequate antenatal recognition, and scarce post‑diagnostic counselling forced families to self‑navigate care amid inconsistent provider engagement. Organizationally, high costs prompted reliance on NGOs; limited specialized services, long waits, and therapist burnout constrained individualized therapy. Community factors included service concentration in Karachi, long travel/relocation, financial burden, and pervasive stigma that curtailed social inclusion and lowered parental expectations. Policy gaps included absent DS‑specific clinical/counselling guidelines and poor epidemiologic data; participants prioritized a national registry to enable follow‑up and coordination. Education was a dominant, unmet need restricted by school policies and costs and often overshadowed health concerns. This study emphasizes the urgent need for system-level reforms and coordinated interventions to improve care pathways for children with Down syndrome in Pakistan. These changes are crucial for ensuring equitable access to timely, quality care for affected families, improving health outcomes, and supporting fuller social inclusion.

## Introduction

Down syndrome (trisomy 21) is a chromosomal disorder caused by the presence of an entire copy or a part of a third chromosome 21. Such patients have distinguishing facial traits and experience mild to moderate intellectual disability [1]. According to the United Nations, the estimated incidence of Down syndrome (DS) is between 1 in 1,000–1 in 1,100 live births worldwide, and each year, approximately 3,000–5,000 children are born with this chromosome disorder [2]. **P**reviously the data on people with disabilities calculated by Pakistan’s government was underreported because not all specially abled individuals were included in reporting. One of the key causes for the inaccuracy of the numbers about people with disabilities is disagreement on the definition of ‘disability’ initially and the refusal of respondents who, in many circumstances, did not want to reveal the disabilities of their children [3] For Down Syndrome, a major contributor, the statistics are similarly less authentic. In a research study conducted in Lahore, Punjab (2005) the prevalence of Down syndrome (DS) was examined. The findings revealed that 1 in 300 babies were diagnosed with DS within the specific cohort [4].

Raising a child with Down syndrome involves unique challenges due to associated health and developmental needs, requiring structured, condition-specific care pathways across multiple domains [3,5].

Nearly half of infants with Down syndrome are affected by congenital heart disease (CHD), the most common congenital anomaly, necessitating structured perioperative and lifelong follow-up care [6,7]. Critical components of this pathway include early surgical intervention, vigilant monitoring for pulmonary hypertension, and a coordinated transition from pediatric to adult cardiac care [8].

Thyroid dysfunction is another common concern in children with Down syndrome, with hypothyroidism occurring more frequently than in the general population [9]. Clinical guidelines recommend annual screening of thyroid-stimulating hormone (TSH) and thyroxine (T4) levels starting in infancy to detect subclinical hypothyroidism early. Screening within the first 1–3 months of life is advised to prevent missed diagnosis, as early signs can be subtle [10].

Children in this cohort may also experience generalized anxiety, specific phobias, or obsessive-compulsive behaviors [11]. These symptoms can be challenging to diagnose due to communication barriers and overlapping characteristics with other conditions [12]. Psychological assessment and support are required as part of routine care for timely intervention to promote emotional stability and healthy development.

Children with Down syndrome typically have motor and language needs [13].In addition to clinical interventions they require multidisciplinary therapies such as occupational therapy for fine motor and self-care skills, physical therapy to address hypotonia and balance deficits, and speech-language or feeding therapy targeting oral motor challenges and safe swallowing [14,15].

While parents in high-income and upper middle-income countries may have better resources and be more accustomed to raising children with special healthcare needs, they still face distinct challenges that require attention. In some contexts, parents perceive healthcare providers as hesitant or disinterested, prompting them to take on complex care tasks themselves, such as managing intricate medication regimens [16] This distrust in the system often leads to gaps in provider–family relationships, leading to missed follow-up appointments [17]. Negative past experiences, including perceived medical errors, further discourage some parents from seeking follow-up care, fearing potential harm to their child [18]. When considering individuals with Down syndrome as a cohort, significant disparities in access to healthcare become evident. In the United States, nearly 20% of this population face substantial geographic barriers to accessing specialty care, a challenge that is particularly pronounced in the Southern states, where over one-third must travel more than two hours to reach the nearest clinic [19].

Health care delivery issues in low- and middle-income countries (LMICs) are distinct and multifaceted. Unlike in high-income nations, Down syndrome screening is uncommon, leading to delayed diagnosis, sometimes until age seven, which adversely affects health outcomes [20].Literature suggests that parents from this region face significant challenges when accessing healthcare, often resulting in frustration due to inadequate communication, lack of information, and perceived incompetence among healthcare providers [21]. Studies have shown that parents frequently seek information, focus on their children’s healthcare needs, and develop support networks to navigate the complexities of care. However, communication gaps and insufficient responsiveness from healthcare professionals often undermine the effectiveness of these strategies [22]. Barriers to rehabilitation services also emerge, with parents reporting communication gaps between staff and families, and a lack of individualized care, leading to misunderstandings and negative perceptions. These challenges are particularly pronounced in profit-oriented organizations that overlook the specific needs of children, reducing the effectiveness of rehabilitation services [23].

### Pakistan and Down syndrome

Individuals with Down syndrome (DS) in Pakistan navigate a healthcare system that is fragmented, under-resourced, and inconsistently inclusive for persons with disabilities [20]. Despite the presence of policy frameworks such as the *National Policy for Persons with Disabilities (2002)* and the *Pakistan Persons with Disabilities Act (2020)*, implementation remains weak, resulting in limited access to comprehensive and coordinated care [24,25].

Systemic obstacles include inadequate resources, lack of provider training, non-standardized documentation, absence of quality assurance frameworks, and minimal health insurance coverage forcing families to self-finance care [20]. These structural gaps are compounded by sociocultural barriers: stigma, financial constraints, and low parental awareness, often evidenced by myths and hidden children. A local study reported that parents frequently perceive DS as a consequence of personal deeds, leading to guilt and social isolation. Such factors impede early diagnosis, therapeutic interventions, and community inclusion [26,27].

Early childhood interventions such as physical, occupational, and speech therapies for this special cohort are largely centralized in urban non-profits like the Karachi Down Syndrome Program (KDSP) and Down Syndrome Club Pakistan [28,29]. However, these services lack nationwide coverage and integration with public health systems.

Diagnostic and clinical pathways remain limited, with significant cost implications. For instance, a tertiary-care study from Hayatabad Medical Complex, Peshawar found that 63% of children with DS had congenital heart disease, highlighting the need for early cardiac screening to reduce downstream disease burden [30]. Initiatives like the DS-Pak registry at the Aga Khan University, Pakistan aim to strengthen continuity of care, they remain nascent in scope and scale [20].

Despite global evidence on DS care models, there is limited research exploring the lived experiences of caregivers and healthcare providers in Pakistan. Existing literature does not adequately capture how systemic fragmentation, sociocultural stigma, and resource constraints shape care pathways. Without national-level integration combining policy enforcement, accessible infrastructure, provider training, data-driven referrals, and sustainable funding the healthcare system continues to fall short of delivering cohesive, lifelong, and holistic care for individuals with DS.

This study seeks to address these gaps by examining stakeholder perspectives, identifying priority areas for specialized healthcare services, and informing policy and practice for improved DS care delivery in Pakistan.

## Materials and methods

### Study design

An exploratory qualitative research design (EQD) was employed to generate an in-depth understanding of context-rich, non-numeric data relevant to complex healthcare experiences [31]. Qualitative methods are well suited to examining lived experiences, health behaviors, and healthcare needs, particularly in settings where empirical evidence is limited and care pathways are not clearly defined [32].

This design is especially appropriate in Pakistan’s context, where down syndrome care pathways are fragmented and shaped by sociocultural and health-system factors [33]. An exploratory approach enables the perspectives of caregivers and healthcare providers to emerge organically, supporting a comprehensive understanding of barriers and facilitators to care.

In-depth interviews (IDIs) were conducted to allow participants to articulate their experiences in detail, facilitating the identification of key challenges, enabling factors, and priorities related to access and quality of services, in alignment with the study’s exploratory aims

### Study setting

The research was carried out at a Non-Profit Organization (NGO) and a private tertiary care hospital located in Karachi, Pakistan.

Karachi Down Syndrome Program (KDSP), founded in Karachi in March 2014 by concerned parents and advocates, is a non-profit organization dedicated to promoting acceptance and inclusion of individuals with down syndrome. The organization serves over 1,700 families nationwide, providing therapy through in-house professionals, including speech, occupational, physiotherapists, and behavioral therapists. Additionally, pediatricians conduct subsidized or free consultations on a bimonthly basis [34].

Due to limited infrastructure for specialized care, the NGO collaborates with hospitals, including the Aga Khan University Hospital (AKUH), a private tertiary care facility established in 1985. AKUH serves as a one-stop solution for children with special needs by offering Down syndrome screening and treatment, as well as providing multidisciplinary care. Individuals from both the NGO and the hospital were interviewed for this study [34,35].

The objective of involving stakeholders from both organizations was to gather a diverse range of data. The inclusion of participants with diverse socioeconomic backgrounds and employment situations, particularly among healthcare providers, improved the research’s validity and depth.

### Study population

In the study, two distinct participant groups were interviewed inclusive of both caregivers and healthcare providers of children with DS. This selection was supported by literature, which shows that combining caregiver and provider viewpoints is critical for identifying resource gaps and achieving integrated, patient-centered care [36].

- *Caregivers (mothers and fathers):* Individuals with lived experience of caring for a child with Down syndrome, offering viewpoints into challenges, support needs, and sociocultural influences on care.
- *Healthcare professionals* (therapists and paediatricians): Providers directly involved in service delivery, who understand systemic and organizational dynamics impacting access, quality, and continuity of care.

This approach not only adds rigor through triangulation but also allows the reader to view the issue from multiple perspectives, essentially seeing both sides of the mirror. Caregivers shared their personal journeys, the emotions, challenges, and decisions they come across while healthcare providers offer their perspective on diagnosing and supporting these families within the constraints of the system.

### Participant eligibility

The inclusion criteria for this study included individuals who were residents of Karachi and were responsible for the care of children between the ages of 0–18. Additionally, they needed to express willingness to be a part of the study. Healthcare providers had to have prior experience in treating children with Down syndrome and resided in Karachi.

### Recruitment strategy

Participants were recruited after obtaining ethical approval from the Aga Khan University Ethical Review Committee (ERC), and data collection was conducted between 11 and 30 August 2023. A ***purposive sampling*** technique was employed to select participants from a non-governmental organization and a private tertiary care hospital. Purposive sampling, widely used in qualitative research, involves identifying individuals who are especially knowledgeable or experienced with the phenomenon of interest [37].

Caregivers were recruited from the waiting areas of participating organizations, while healthcare providers were contacted via email to schedule interviews at their workplace

### Reflexivity

This study was conducted as part of the principal investigator’s master’s degree at the Aga Khan University (AKU), Pakistan. The multidisciplinary research team included expertise in health systems research, Pediatrics, nursing, qualitative methods, and health services research.

The PI is a health systems researcher at AKU, led the study design, data collection, and initial coding and analysis, with a focus on understanding how health service structures shape caregiver experiences. A pediatric geneticist (Professor and Chair, Women and Child Health Division, AKU, and Director of the Karachi Down Syndrome Program) informed interpretation of care pathways and referral systems for children with Down syndrome. His experience working with families and community organisations helped interpret caregivers’ and healthcare provider narratives within the broader service delivery context.

A pediatric nurse and nursing researcher (Associate Professor and Associate Dean, School of Nursing and Midwifery, Aga Khan University) brought expertise in family-centred care and child health services, which supported interpretation of caregiver–provider interactions and barriers encountered when navigating health services. A qualitative research expert and social scientist (Assistant Professor, Department of Community Health Sciences, Aga Khan University) ensured methodological rigour and guided the analytical process, drawing attention to participants’ narratives and social contexts shaping access to care. A health services researcher (Assistant Professor, Department of Community Health Sciences, Aga Khan University) contributed expertise on health systems organisation and policy environments, supporting interpretation of institutional and system-level factors influencing service delivery.

Guided by the socio-ecological framework, the team interpreted findings across multiple levels. Clinical perspectives helped contextualise individual and family experiences within existing care pathways, while the qualitative researcher emphasised participants lived experiences and social contexts. The health services expert supported interpretation of institutional and policy-level influences shaping service access and coordination. Coding and theme development were discussed collaboratively with the research team to validate interpretations, and reflexive notes documented assumptions and analytical decisions. This multidisciplinary perspective strengthened the analysis by situating caregiver and provider experiences within the broader health system and social environment.

### Data collection and management

Semi-structured interview guides were specifically tailored for each category (caregiver and healthcare provider) for the purpose of this study. Each interview guide consisted of 7–10 open ended questions which were adapted and developed after formative discussions with the team and literature review in the view of a socio-ecological Model (SEM). The interviews were conducted in both English and Urdu catering to the language preference of the study participants. Following the interviews, the recorded conversations were then transcribed and later translated into English by the PI. In addition, short Field notes taken throughout the interview process served as valuable references providing the team with a deeper understanding of the participant “quotes”.

Informed consent was obtained from all participants, who were given the option to be audio recorded during the interviews. Signed consent forms were then securely stored under lock and key while the interview recordings were kept on a password-protected device. Each participant was given an estimated 30–45 minutes for each interview. Prior to conducting interviews, suitable times and locations were arranged for each participant. Informed consent was obtained from all participants, who were given the option to be audio recorded during the interviews.

### Data analysis

**Fig 1:**
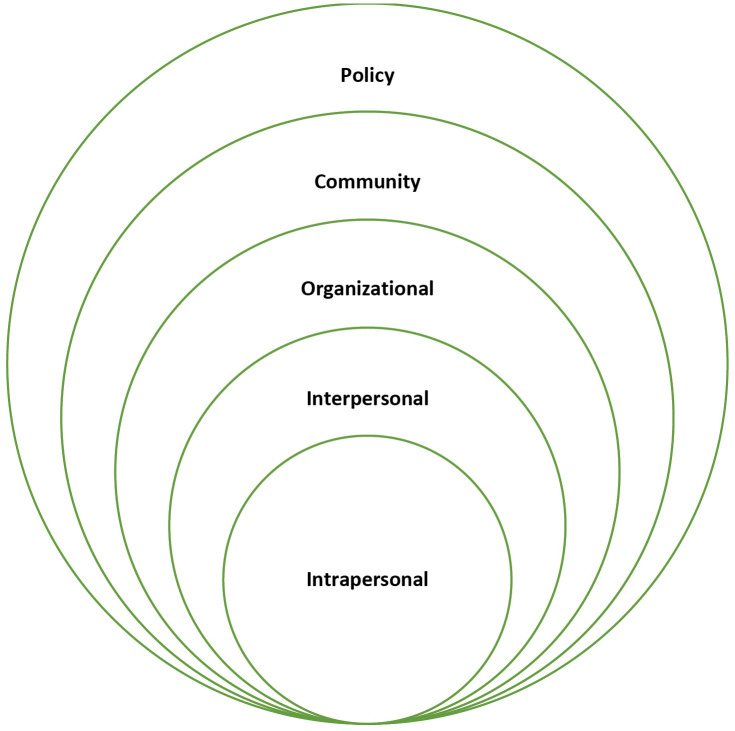
Socio-ecological model of health.

A deductive content analysis approach was employed within the framework of a socio-ecological model. The socioecological model (SEM) (**Fig 1**) provides a multi-level framework for understanding health behaviours, recognizing that individual outcomes are shaped by interactions across personal, social, organizational, community, and policy contexts [38,39]. At the individual level, factors included participants’ coping strategies following diagnosis. The interpersonal level reflected caregivers’ knowledge and their interactions with healthcare providers. Organizational influences involved resources and the financial implications of seeking care. Community-level factors highlighted cultural attitudes, geographic barriers, and social stigma. Finally, policy-level determinants included national health guidelines and institutional protocols shaping access to services. SEM is extensively employed in qualitative research to examine how marginalized populations utilize healthcare, their requirements, obstacles, and their overall experiences [40].

The selection of this framework was a result of collaborative input from the research team members to ensure a well-informed research design. Recurring patterns in the data were identified and coded by the principal investigator in accordance with predefined themes guided by the Social Ecological Model (SEM) framework (Table 1). Coding was supported by Atlas.ti software to enhance organization, consistency, and analytical rigor. All codes and emerging themes were systematically reviewed and validated through regular analytical discussions with the study supervisor and co-supervisor. These discussions involved revisiting coded transcripts and reaching consensus on theme development to strengthen the credibility and confirmability of the analysis [41]. Any inconsistencies identified during the analytical process were addressed through triangulation and collaborative review, ensuring validation of narratives across different participants and maintaining the integrity of the findings.

**Table 1:** Thematic Map.

Themes guided by SEM	Sub Themes	Examples of Quotations
*Intrapersonal Factors*	**Emotional Impact of the Diagnosis**	*“This was the longest phase of your life where you keep thinking it can’t be true because you’re not a high-risk patient to have a child with DS. You’re not in that age, you didn’t get married to your cousin, you don’t have a family history of Down syndrome then why me?”*
		“ *The non-antenatally diagnosed, they found it very difficult initially to digest that there is something wrong or maybe not wrong, something different with their child.”*
		*“Parents perceive it as a disease and ask if it can be cured.”*
*Interpersonal Factors*	**Delays in Diagnosis**	*“I had many anomaly scans during my pregnancy… we were worried that the child may be preterm, but nothing prepared us for Down syndrome.”*
		*“I, being a therapist, can identify a child with DS, so a doctor should not lack.”*
		*“We are lacking a lot of antenatal scans and antenatal visits for sure... whoever is doing the antenatal scan, they aren’t looking for this.”*
	**Lack of Appropriate Medical Counselling**	*“He is a Mongol... the doctor said this and went away. He didn’t tell me what it was.”*
		*“The doctor didn’t tell us from where to go after the diagnosis which direction to follow. She used to stay sick often... I have raised her with a lot of difficulty”*
	**Disparities in Provider Engagement and Support**	*“Some doctors act indifferent; they act as if my child is not worth it.”*
		*“No one took notice for a very long time... she had bad eyesight, very bad eyesight.”*
*Organizational Factors*	**Cost of Healthcare and Reliance on Non-Governmental**	*“Actually, the area we live in is lower class; these children are left on their own We however did run for our child. If we went elsewhere other than this NGO, we wouldn’t be able to do it because it would be costly there and everything has been made easier here.””*
		*“Most parents pay out of their pocket, and they tend to skip on Echo, thyroid testing or therapies*
	**Limited Resources**	*“There are no such setups here to deal with our children’s problems. I mean when we go to other doctors, they don’t know what problems they may have”.*
		*“This NGO is an excellent resource but is located far away, so we cannot cater to people living in all parts of Karachi.”*
	**Quality Improvement to Navigate Therapist Burnout**	*“Reduction of work burden is needed. But 8 sessions a day should be reduced to 5 or 6 so I can do more qualitative work”*
		*“There should not be more than 7 to 8 sessions, but this is not taken into account. Working six days a week without a break affects our work and mental health.”*
Community Factors	**Geographic Barriers to Access**	*“......I took the initiative to look for KDSP. I got him entered into the program on the phone. We lived in Sukkur. There was nothing for my child over there. KDSP was famous. We moved to Karachi for his sake”*
		*“...... In Pakistan there is no other institute working with children having down syndrome apart from KDSP in terms of various services inclusive of healthcare. In Rehabilitative departments other comorbid conditions are not referred continuously and do not provide quality services. If you remove KDSP there is nothing in Pakistan.”*
	**Social Barriers and Cultural Stigmatization**	*“Husband thinks that wife did something; wife thinks that it was because of a solar or lunar eclipse, so because of that Down syndrome has occurred. There are so many ridiculous myths!”*
		*“Parents of normal kids look at us as if we have taken “tamgha-e-Imtiaz “. Firstly, we are not Bechara and secondly these children are not a burden*
Policy Factors	**Need for Specialized Guidelines**	*“I follow my own pattern; there are no guidelines. We provide counselling based on what needs to be conveyed to the family. Every family is different”*
		*“There are guidelines for hypothyroidism, but for the management of hypothyroidism in Down syndrome, there is no specific guidelines”*
	**Deficiency of Data for Down Syndrome**	*-“................what matters is that we should have a registry, and people should know about it and then every baby with Down’s syndrome should get enrolled.”*
		*“I’m not aware of the stats, but the last article I read stated that every 1 in 100 child is born with DS in Pakistan’s population”*
	Educational Needs	*“My son is six years old. We haven’t been able to get him admitted anywhere. We don’t have enough income to take him to a mainstream school”*
		*“What makes it difficult is that instead of focusing on their health, initial challenges they are more concerned on his education and till what grade will the child be able to study and if she can ever get married or not. We do counsel them but its continuous process”*

### Ethical considerations

The study protocol was approved by the Ethical Review Committee (# 2023-8821-25951) of Aga khan university Karachi, Pakistan. Consent forms were also approved by the board and included a detailed description of the study in a language easily understood by the participants. Since KDSP does not have its own ethical review committee, their internal department reviewed the interview guide to ensure alignment with organizational policies and ethical protocols.

To ensure confidentiality in the research, each participant was given a unique ID and data was stored in a password protected device only accessible to the research team. This ensured confidentiality of this research and their participation in it. The Consent forms also gave all participants the right to refuse participation in this research. Data was not shared with any individuals outside the research team, and the participants were made aware that there were no financial incentives to the research prior to their agreement to be interviewed.

## Results

In this qualitative study, 23 in-depth interviews (IDIs) were conducted with caregivers and healthcare providers to explore their experiences and perceptions regarding Down syndrome (DS) diagnosis and care. Interviews were held at participants’ chosen facilities and times, averaging 30–45 minutes each. Data saturation was achieved after 20 interviews; however, three additional interviews were conducted to enhance the robustness of the findings. Notably, most caregivers identified as middle or upper-middle-class individuals. Participant details are presented in Table 2 below:

**Table 2:** Participant Characteristics.

Participant Type	N	Education/Specialty	Age Range
Mothers	8	Matric to Master’s	26–60 yrs
Fathers	2	Intermediate, MBBS	36–42 yrs
Therapists	8	Occupational, Physio, Speech-Language, Behavioral	22–33 yrs
Pediatricians	5	Developmental, General, Neonatal, Endocrinology	35–42 yrs

### Theme 1: Intrapersonal factors

#### 1.1 Emotional impact of the diagnosis.

Caregivers, particularly mothers, recalled deeply emotional experiences following their child’s diagnosis. Many reported progressing through grief like trajectories, including denial, anger, and depression, before eventually reaching a point of acceptance. The diagnosis, especially when involving a firstborn, was often met with initial shock. However, over time, many caregivers came to view their child’s condition as part of Allah’s will, shifting their focus toward offering consistent and unconditional support.

*“This was the longest phase of your life where you keep thinking it can’t be true because you’re not a high-risk patient to have a child with DS. You’re not in that age, you didn’t get married to your cousin, you don’t have a family history of Down syndrome then why me?*

*-Mother, IDI-CG-III.*

*“I said okay! Shukar Alhamdulilah. Whats next?”*

*-Mother, IDI-CG- VI*

Healthcare providers corroborated these reactions, observing parental shock and reluctance to accept the diagnosis.

*“Parents perceive it as a disease and ask if it can be cured.*

*-Pediatrician, IDI-HCP-IV.*

A health care provider from the private hospital stressed the significance of noting that parents who receive diagnosis during pre-natal screening generally find it more manageable to accept the news. A healthcare provider recounted his experience recalling that:

“ *The non-antenatally diagnosed, they found it very difficult initially to digest that there is something wrong or maybe not wrong, something different with their child.”*

*-Neonatologist, IDI-HCP-III.*

### Theme 2: Interpersonal factors

#### 2.1 Delays in diagnosis.

Caregivers frequently described significant delays in receiving a diagnosis of their child’s Down syndrome, often attributing these delays to healthcare providers. These delays, ranging from birth to as late as three years, contributed to feelings of neglect and frustration among families. Many caregivers reported that despite undergoing routine prenatal scans, no concerns were communicated to them.

*“I had many anomaly scans during my pregnancy… we were worried that the child may be preterm, but nothing prepared us for Down syndrome.”*

*-Mother, IDI-CG-VIII*

“*The doctors showed some doubts about his health, but no concerns or reports were shared with us”*

*-Mother, IDI-CG-IV*

Healthcare providers, particularly therapists and pediatricians, acknowledged these diagnostic delays. They cited gaps in antenatal screenings and a general lack of attention to key indicators during early assessments. These shared perspectives point to systemic gaps in early identification and communication within maternal and child healthcare services.

*“I, being a therapist, can identify a child with DS, so a doctor should not lack.”*

*-Physiotherapist, IDI-HCP-V*

*“We are lacking a lot of antenatal scans and antenatal visits for sure... whoever is doing the antenatal scan, they aren’t looking for this.”*

*- Pediatrician, IDI-HCP-II*

#### 2.2 Lack of appropriate medical counseling.

Many caregivers expressed emotional distress and frustration over the lack of clear information provided after their child’s Down syndrome diagnosis. Several reported that medical staff offered little to no explanation, leaving families confused and unprepared to manage the condition. This lack of guidance often forced caregivers to seek information on their own, often leading to feelings of anxiety and isolation. In some cases, families even relocated in search of better support and counseling services. The absence of structured post-diagnostic counseling emerged as a significant gap in care, highlighting the need for improved communication and family-centered support systems.

*“He is a Mongol... the doctor said this and went away. He didn’t tell me what it was.”*

*- Mother, IDI-CG-V*

*“In foreign countries, if a child is born with special needs there are counseling sessions for the parents also. Here there is no counseling.”*

*- Mother, IDI-CG-I*

“*The doctor didn’t tell us from where to go after the diagnosis which direction to follow. She used to stay sick often... I have raised her with a lot of difficulty”*

*-Mother, IDI-CG-IX.*

#### 2.3 Disparities in provider engagement and support.

Caregivers reported mixed experiences with healthcare providers following their child’s diagnosis of Down syndrome. While most of them believed that their child was treated fairly like any other patient, their experiences varied from one healthcare provider and medical facility to another. A mother who is a regular at the NGO and the private hospital explained that

*“Some doctors act indifferent; they act as if my child is not worth it.”*

*-Mother, IDI-CG-VI*

Caregivers emphasized the importance of being guided by providers not only in understanding the diagnosis but also in navigating available services. These inconsistencies across healthcare facilities often left families feeling unsupported and frustrated.

*“The doctors should guide us. Tell us what we can do! Educate us about what services are available. Tell us about therapies, reach out to the parents and give them directions”*

*-Mother, IDI-CG-III*

In some cases, perceived indifference had tangible consequences for the child’s health, highlighting the need for more consistent and informative care across settings.

*“No one took notice for a very long time... she had bad eyesight, very bad-eyesight.”*

*-Mother, IDI-CG-VIII,*

### Theme 3: Organizational factors

#### 3.1 Cost of healthcare and reliance on non-governmental organizations.

Financial barriers emerged as a major barrier in accessing essential services for children with Down syndrome. Caregivers, particularly from lower-income backgrounds, described the high cost of care as a limiting factor, often relying on NGOs for support due to the lack of affordability of private services.

“*Actually, the area we live in is lower class; these children are left on their own We however did run for our child. If we went elsewhere other than this NGO, we wouldn’t be able to do it because it would be costly there and everything has been made easier here.”*

*-Father, IDI-CG-II.*

Many caregivers expressed distrust toward government institutions, viewing them as inadequate or inaccessible for meeting their children’s needs. They unanimously believed that obtaining therapy and addressing other healthcare concerns is challenging, primarily because healthcare in Pakistan is expensive and government organizations are perceived as unreliable.

*“The government institutes are not an option for us because of the stories I have heard about them. There is nothing for our child from the government”*

*-Mother, IDI-CG-III*

Healthcare providers confirmed these concerns, acknowledging that families often forego important diagnostic tests and therapies due to out-of-pocket expenses. Socioeconomic disparities were seen as a key determinant of access, with professionals noting that financial means significantly influenced the quality and continuity of care families could secure.

*“Most parents pay out of their pocket, and they tend to skip on Echo, thyroid testing or therapies.”*

*— Pediatrician, IDI-HCP-II*

*“Having money will give accessibility to everything. The lower middle class aren’t so lucky.”*

*— Speech-language therapist, IDI-HCP-VII*

#### 3.2 Limited resources.

Both caregivers and healthcare providers highlighted the limited availability of specialized care and trained professionals for children with Down syndrome. Families reported significant challenges in accessing appropriate services, often due to a lack of dedicated facilities, long waiting times, and geographic barriers. These limitations placed additional strain on caregivers, especially those from underserved areas.

“*There are no such setups here to deal with our children’s problems. I mean when we go to other doctors, they don’t know what problems they may have”.*

*— Mother, IDI-CG-V*

***“….*** *the required services were not available for the larger portion of the public. Our family position made us reach the doors of this private hospital, however not every family could make to that****”***

*— Father, IDI-CG-VII*

Healthcare providers acknowledged these systemic gaps, noting that while some NGOs offer high-quality support, their reach is limited. The scarcity of inclusive, city-wide services reinforced the sense of isolation and inadequacy in the current healthcare infrastructure.

*“This NGO is an excellent resource but is located far away, so we cannot cater to people living in all parts of Karachi.”*

*— Physiotherapist, IDI-HCP-VIII*

*“This NGO is like a bubble... There is nothing outside this bubble for these kids.”*

*— Developmental pediatrician, IDI-HCP-I*

#### 3.3 Quality improvement to navigate therapist burnout.

An emerging theme across both participating institutions was concern regarding high caseloads and the limited availability of trained therapists, and how these factors affected the quality and sustainability of therapy services for children with Down syndrome. Therapists reported that excessive patient volumes constrained individualized attention and tailored interventions, while also contributing to emotional and physical strain among providers.

“*Reduction of work burden is needed. But 8 sessions a day should be reduced to 5 or 6 so I can do more qualitative work*”

— Occupational therapist, IDI-HCP-VI

*“Our first preference is given to children in Karachi. Even they have to wait months to start therapy with us.”*

*— physiotherapist, IDI-HCP-VIII.*

Reducing the number of daily therapy sessions was viewed not only as a means to improve therapeutic outcomes but also as important for mitigating work-related stress and preventing burnout in a context where a small number of therapists were expected to meet growing service demands. Therapists described prolonged work schedules, limited rest, and sustained emotional demands as factors affecting their well-being and capacity to deliver effective care.

“There should not be more than 7 to 8 sessions, but this is not taken into account. Working six days a week without a break affects our work and mental health.”

— Speech language therapist, IDI-HCP-X

### Theme 4: Community factors

#### 4.1 Geographic barriers to access.

Specialized services for children with Down syndrome are primarily concentrated in major urban centers such as Karachi, with limited or no availability in smaller towns or rural areas. Families from outside the city report traveling long distances or relocating to Karachi to access therapy and medical services. However, even after relocation, families experience prolonged waiting times for therapy initiation due to high service demand and limited capacity.

*“......I took the initiative to look for KDSP. I got him entered into the program on the phone. We lived in Sukkur. There was nothing for my child over there. KDSP was famous. We moved to Karachi for his sake”*

*-Mother, IDI-CG-III*

*“...... In Pakistan there is no other institute working with children having down syndrome apart from KDSP in terms of various services inclusive of healthcare. In Rehabilitative departments other comorbid conditions are not referred continuously and do not provide quality services. If you remove KDSP there is nothing in Pakistan.”*

*- occupational therapist, IDI-HCP-IX*

Families who rely on public transport describe daily travel to therapy centers as time-consuming and financially burdensome, particularly in the context of children’s behavioral and emotional needs.

*“It takes me 40–45 minutes to travel every day for therapies. Parents who are coming on public transport face a lot of issues. These children are very temperamental; they lose their temper anytime, plus the expense of commuting to KDSP.”*

*— Mother, IDI-CG-X*

Despite these challenges, caregivers report observable improvements in their child’s health status after accessing specialized services following relocation.

*“She has started keeping well since we moved from Abbottabad… She has no health issues anymore.”*

*— Mother, IDI-CG-IX*

#### 4.2 Social barriers and cultural stigmatization.

Cultural stigmas surrounding Down syndrome in Pakistan lead to social exclusion and discrimination against affected individuals and their families. Prevailing misconceptions and myths contribute to negative attitudes, resulting in limited acceptance within communities.

*“Husband thinks that wife did something; wife thinks that it was because of a solar or lunar eclipse, so because of that Down syndrome has occurred. There are so many ridiculous myths!”*

*- Neonatologist, IDI-HCP-III*

*“Parents of normal kids look at us as if we have taken “tamgha-e-Imtiaz “. Firstly, we are not Bechara and secondly these children are not a burden”*

*-Mother, IDI-CG-X*

Participants emphasized the pervasive societal stigma surrounding Down syndrome, which significantly limited social integration for affected children. Healthcare providers further observed that this stigma shaped parental perceptions of their children’s potential.

“*Our society has made DS a topic of taboo. No one talks about it and no one knows what it is.”*

*-Mother, IDI-CG-IV*

*“...the parents, the mother feels like the children are of no use!....... What we (therapists) do is a waste of time these children cannot accomplish anything….”*

*- occupational therapist, IDI-HCP-IX*

### Theme 5: Policy factors

#### 5.1 Need for specialized guidelines.

Healthcare providers reported the absence of clear, standardized guidelines for the management and counseling of children with Down syndrome, noting that clinical practices were largely shaped by individual judgment and experience. Providers described adopting personalized approaches to counseling and intervention, tailored to the perceived needs of each family rather than guided by formal protocols

“*I follow my own pattern; there are no guidelines. We provide counseling based on what needs to be conveyed to the family. Every family is different”*

*- Developmental Pediatrician, IDI-HCP-I*

Subspecialists similarly highlighted gaps in condition-specific guidance, with a healthcare provider noting that

*“There are guidelines for hypothyroidism, but for the management of hypothyroidism in Down syndrome, there is no specific guidelines”*

*- Pediatric endocrinologist, IDI-HCP-XIII*

#### 5.2 Deficiency of data for down syndrome.

Healthcare providers reported limited awareness of available data on the prevalence of Down syndrome in Pakistan. During interviews, several providers indicated that they were not familiar with current statistics and, in some cases, referred to approximate figures or older sources of information. One provider stated,

*“I’m not aware of the stats, but the last article I read stated that every 1 in 100 child is born with DS in Pakistan’s population”*

*Occupational Therapist, IDI-HCP-VI.*

Considering this limited awareness, healthcare providers suggested the need for a centralized registry to systematically record, and track children diagnosed with Down syndrome. Providers described such a registry as a practical mechanism to support follow-up and continuity of care. They explained that:

*- “There is a need for a registry so we can retrieve them and reach out to them; otherwise, patients will be lost”*

*Paediatrician, IDI-HCP-IV.*

A neonatologist similarly emphasized the importance of early enrolment, stating that

*-“................what matters is that we should have a registry, and people should know about it and then every baby with Down’s syndrome should get enrolled.”*

*Neonatologist, IDI-HCP-III.*

#### 5.3 Educational needs.

Access to quality education emerged as a major concern for caregivers, who often viewed it as equally important as access to healthcare. Financial difficulties and rigid school policies made it even harder to find suitable educational options for children with Down syndrome. One mother shared:

*-“My son is six years old. We haven’t been able to get him admitted anywhere. We don’t have enough income to take him to a mainstream school”*

-*Father, IDI-CG-II.*

*-“Education is the major barrier for us. As for health it’s not possible in Pakistan to do anything for these children. people who have money things are easier but not for us”*

*-Mother, IDI-CG-VIII*

Healthcare providers acknowledged the emotional and practical burden this placed on families, observing that worries about education often took precedence over health-related concerns.

*-“What makes it difficult is that instead of focusing on their health, initial challenges they are more concerned on his education and till what grade will the child be able to study and if she can ever get married or not. We do counsel them but its continuous process”*

*-Clinical Psychologist, IDI-HCP-XI*

These sentiments pointed towards broader gaps in the education system’s ability to meet the specific needs of children with Down syndrome.

## Discussion

This study explored the experiences of caregivers of children with Down syndrome (DS) in Pakistan, alongside insights from healthcare providers, to develop a comprehensive understanding of the healthcare landscape for individuals with DS. Guided by the socio-ecological framework, findings were analysed across intrapersonal, interpersonal, organizational, community, and policy levels.

At the intrapersonal level, caregivers described strong emotional reactions following their child’s Down syndrome diagnosis, moving from shock and denial to gradual acceptance. These emotional trajectories were often shaped by cultural and religious beliefs, which influenced how caregivers understood and coped with the diagnosis. Similar emotional distress and coping patterns have been reported internationally, including studies from Ireland [42] and Kuwait [17], where parents described comparable emotional responses regardless of whether the diagnosis was made prenatally or postnatally. However, in Pakistan’s context, these experiences are embedded within strong religious and familial belief systems that shape how disability is interpreted within families. Literature suggests that religious beliefs frequently help caregivers make meaning of the diagnosis, for example by framing it as “God’s will” or a “test of faith”, which supports emotional regulation and acceptance [43]. At the same time, these interpretations may also interact with prevailing social expectations around motherhood and family responsibility, which can intensify feelings of guilt, blame, or fear of social judgment within extended family and community settings [44,45]. This suggests that caregivers’ emotional adjustment is not only psychological but culturally mediated, highlighting the importance of counselling and support services that acknowledge and engage with religious and cultural belief systems rather than treating them as peripheral influences.At the interpersonal level, significant gaps were identified in counselling, communication, and emotional support following a child’s Down syndrome diagnosis. Caregivers described receiving unclear or minimal guidance at diagnosis, which contributed to confusion, emotional distress, and delayed care-seeking. These experiences reflect evidence from Pakistan showing that healthcare providers often lack training in delivering diagnosis sensitively and may use inappropriate or outdated terminology, pointing to broader gaps in communication skills and provider preparation [26]. In Pakistan’s health system, where physician training often prioritizes clinical management over communication and family counselling [46], such gaps can significantly influence how families interpret and respond to a diagnosis. International literature similarly shows that poor information provision increases caregiver stress and limits families’ ability to navigate care pathways effectively [16,47,48], but in contexts such as Pakistan where alternative sources of reliable information are limited, the consequences for care-seeking decisions may be even more pronounced.

Disparities in empathy across healthcare providers further shaped caregivers’ experiences. While some families felt supported, others perceived indifference or lack of engagement, which in some cases led them to seek care elsewhere. Findings from this study suggest that such empathy gaps may stem from structural constraints such as high workload, limited consultation time, and absence of structured post-diagnostic counselling rather than intentional neglect [49] In Pakistan’s resource-constrained healthcare settings, particularly in high-volume public or private hospitals, providers often manage large patient loads with limited multidisciplinary support [50]. As a result, healthcare providers’ focus on clinical tasks, combined with limited training in family-centered communication and Down syndrome–specific needs, may reduce their capacity to address caregivers’ emotional concerns. Similar patterns of caregiver marginalization within healthcare systems have been reported in other settings [16,51]. Together, these findings highlight that improving empathy and support requires not only individual provider effort but also system-level changes, including training, time allocation, and standardized counselling pathways.

At the organizational level, the financial barriers identified in this study highlight the need for system-level responses to reduce the economic burden on families of children with Down syndrome. Healthcare providers reported that high costs frequently led parents to forgo essential diagnostic tests, a concern echoed by caregivers who often relied on NGO support due to limited public-sector options. In Pakistan, where healthcare financing is largely dependent on out-of-pocket expenditure and specialized developmental services are concentrated within the private sector, families often bear substantial financial responsibility for diagnostic and therapeutic care [52,53]. These findings are consistent with evidence from comparable settings, where families incur substantial out-of-pocket expenses, with up to 33% experiencing severe financial strain and 46% resorting to borrowing to meet care needs [54]. Together, this highlights the inadequacy of existing public healthcare services in addressing the complex and long-term needs associated with Down syndrome. System-level measures such as subsidized diagnostic testing, integration of Down syndrome–related services into public maternal and child health packages, and standardized diagnostic and referral pathways may help reduce unnecessary costs and improve access to care.

Community-level factors highlighted cultural stigma as a pervasive barrier reported by both participant groups. Healthcare providers noted that misconceptions and prevailing myths surrounding Down syndrome often undermine scientific understanding and complicated effective counselling interactions for families. In many communities in Pakistan, disability is still associated with social stigma, which may influence how families disclose diagnosis and seek support [55]. Social stigmatization further restricted support networks and opportunities for integration, adversely affecting both parental mental health and children’s educational participation. These findings align with national and global evidence on courtesy stigma, where parents report shame, guilt, and social isolation [55–57]. However, the influence of extended family structures and community perceptions in Pakistan may amplify these experiences, highlighting the need for culturally sensitive counselling and broader public awareness initiatives.

Access to specialized DS services was found to be highly centralized in urban centers, particularly Karachi, leaving families in smaller towns and rural areas with limited options. This resulted in long travel distances, relocations, and prolonged waiting times for therapy initiation. Such patterns reflect broader inequalities in disability and rehabilitation services across Pakistan, where specialized developmental and rehabilitation services remain concentrated in a few urban tertiary centers and NGO-supported facilities [58]. Despite these challenges, caregivers and healthcare providers reported noticeable improvements in children’s health after accessing specialized care, emphasizing the benefits of such services and the inequities created by their limited distribution.

At the policy level, the absence of standardized clinical guidelines for DS management was evident. Care decisions relied on individual experience rather than evidence-based protocols, highlighting an urgent need for national guidelines. Additionally, the lack of a centralized data system limits accurate prevalence estimation and resource planning. In Pakistan, where national disability surveillance systems remain fragmented, this lack of data further constrains policy planning and allocation of specialized resources [20]. Establishing a DS registry, similar to the NIH’s DS-Connect Registry in the United States [59], could facilitate systematic data collection, inform policy development, and optimize resource allocation to improve outcomes for individuals with DS.

Overall, the findings suggest that caregivers’ and healthcare providers’ experiences influencing perceptions of Down syndrome care are shaped by interconnected gaps across multiple socio-ecological levels. Delays in diagnosis, limited counselling, and inconsistent provider engagement reflect broader weaknesses in care coordination, referral pathways, and multidisciplinary practice. These challenges are amplified by financial constraints, geographic concentration of services, and persistent cultural stigma, which together restrict families’ ability to access timely and appropriate support. The absence of standardized clinical guidelines and centralized data systems further limits consistency in care and planning. Taken together, these findings indicate that improving Down syndrome care requires system-level strengthening that integrates clinical guidance, coordinated service delivery, culturally sensitive family support, and improved data infrastructure, rather than isolated interventions focused solely on individual providers or families.

## Strengths and limitations

### Strengths

The study provides context-specific insights from Karachi into Down syndrome care experiences and includes perspectives from two key stakeholder groups, i.e., caregivers and healthcare providers enabling triangulation of experiences. It represents early exploratory work on DS care in Pakistan, highlighting key gaps for future research and service development.

### Limitations

The study sample was limited to participants in Karachi, restricting the generalizability of findings to the wider population.

## Recommendations

Collaborative efforts to record cases of Down syndrome, provide subsidized services at healthcare facilities, and establish centralized, comprehensive support for affected children are essential. Equally important is training medical students and healthcare staff to better recognize the syndrome, addressing a significant gap in care. Raising public awareness through social media campaigns, developing specialized counselling guidelines, and introducing a national screening program are also critical steps to ensure early detection, informed support, and inclusive care for children with Down syndrome and their families.

## Conclusion

Children with Down syndrome and their families face significant challenges, including delays in diagnosis, inadequate medical counselling, financial hardships, limited awareness, and social stigmatization. Addressing these issues requires comprehensive interventions such as specialized counselling guidelines for parents, improved training for healthcare providers, and initiatives to reduce the financial burden on families. Additionally, raising public awareness and establishing a centralized registry are critical steps toward strengthening support systems. Collaborative efforts from healthcare providers, policymakers, and society are essential to ensure equitable access to healthcare and support for children with Down syndrome and their families.

## Supporting information

S1 Data De-identified participant data used for qualitative analysis. (ZIP)
