# Supplementary material for: Experiences of caregivers and healthcare providers regarding health services for children with Down syndrome in Karachi; Pakistan
Source: PLOS Glob Public Health. 2026 Apr 30;6(4):e0006225. doi: 10.1371/journal.pgph.0006225 (PMC13132430; doi:10.1371/journal.pgph.0006225)
Supplement: S1 Data — (ZIP) [file pgph.0006225.s001.zip › Minimal Anonymized Data transcripts/Caregiver-CG2- For PlOS.docx]

After introducing the Principal Investigator (PI) and the research topic to the participant, the consent form was explained in detail. Written consent was then obtained from the participant for both participation in the research and audio recording.

**Participant ID: IDI-CG-XX**

**Date: 28^th^ Aug 2023**

| Can you give me a few details about yourself like name, age, education, marital status, age of your child with ds and socio-economic status (optional)? | (Name XYZ)  Age: 26  Marital status: married  Bachelors in commerce  House wife.  Upper middle class  1 year 10 months (age of child) |
| --- | --- |
| 1. What is your relationship with the child?  - In case of mother/ father probe about the total number of children - In case of sibling probe about the total number of siblings - Do you care for the child alone or do you have help? | Mother.  I have a four-year-old daughter and one younger son with Downs. I have help. My mother-in-law usually takes care of him when I am not around.  Ie two kids |
| 1. Can you tell me at what age was your child diagnosed with Down syndrome?  - Who made the diagnosis? Was it in a clinic or hospital? - Were you aware of what it was? - Did your doctor counsel you about what it was and how to deal with him? - probe about possible questions that may have remained un-answered  1. What was the emotional impact of this news on you and your family? | He was diagnosed the next morning of his birth when the doctor called me. My son (Name of child) was not moving. The doctor came to me by 9.30 am. I told him he’s not moving he simply said “HE IS A MONGOL” he said this statement and went away. He didn’t tell me what it was, he didn’t even give me a clue. I started searching and I found a friend through (name of famous women group) Pakistan and that’s how I reached (name of NGO) when (name of child) was a month old.  Answered above.  No, I didn’t know a thing. I didn’t know if I was standing on the floor. I had no clue, none at all. We don’t have such children in our family.  Since the doctor never briefed us We started asking around. My husband’s friend is a doctor he stated that it can be down syndrome because they mostly use the term Mongol for them but he was not sure.  I remember the doctor and staff had his clothes removed examined him told us he’s a Mongol and left without an explanation. we didn’t realize what was happening,  No counseling. I remember my husband had gone home because of a rough night to sleep and I had called him to tell him what the doctor said.  I didn’t let this get to me after wards, it took time but I did overcome it quickly. I searched if it was curable or not and self-taught myself.  I didn’t understand a few things and there was no one to guide. Then (name of a friend) my Facebook group friend guided me to NGO. They recommended a karyotyping test because they still weren’t sure its downs syndrome because his eyes were the key characteristic and many people in my family had similar eyes  My husband just handed the positive report to me. We didn’t talk about it. we didn’t discuss it with anyone or with each other.  The elderly members in my family did not accept that my child had DS. Neither before the diagnostic test nor after it. After more than 20 months they have started to accept it otherwise total DENIAL mode because my son achieved all his milestones very quickly on time. |
| 1. How often do you visit hospital/ clinics for your child?  - probe about the distance and accessibility - Probe about any specific facility the caregiver visits and does someone accompany him/her? - Do you feel like your child is treated as any other patient during these visits? | We visit the hospital very often. Two visits a month are mandatory. These are general visits and not hospitalizations because he stays sick very often. I used to visit ( name of local hospital) medical it’s a private hospital, but they were not able to judge him because we had to bring him in again and again.  my husband and I go together but it’s only me because my husband’s nature of work forces him to travel outside Karachi.  Yes, yes Ahmadullah. just we wish there was something for our children’s education which is a problem |
| 1. Does your child suffer from any specific health conditions which require for you to see a specialist periodically? (Thyroids, vision/hearing, cardiac related problems, mental health issues, delayed speech etc.?)   Only if the answers to the above question is yes:  Inquire about the type/ name of institute he is visiting and an idea of what kind of services are common for him to on those visits | He used to have a hole in his heart by birth but that hole filled up on his own as per his echo. A valve in his heart is open slightly. We are visiting DR XYZ at private hospital for this and dr XYZ who is a pediatrician.  Dr XYZ (pediatrician) also told me he has issues in his lungs and put him on antibiotics. I am scheduled to see him on the 5^th^ of next month. All these visits are consults and OPD clinics. Nothing major as for now. |
| 1. What challenges did you come across while trying to access healthcare for your child in Karachi?  - probe about structural difficulties in facilities (wheel chairs, elevators or anything that may be of use but wasn’t there) - What are the factors that made getting access to healthcare easier? - Do you feel like your child is getting all the relevant health services he/she may require - probe about the doctor’s behavior and support ? (covered in the statement in the beginning) - probe about waiting time (answered in the beginning)  1. How do you manage to keep up with the financial aspect of getting health services for your child?  - Probe about support money from any organization or otherwise? | We went to different hospitals. None of the doctors referred us to NGO or what to do. We ended up with Dr (name of geneticist) at Private hospital who explained everything in detail. I live in(address) near the NGO. I visit Private hospital and neighborhood health facility. I have no difficulty with that.  Can’t think of much right now we have had no problems. What I mean is we are people who can afford which may be the most important barrier. But I haven’t faced many difficulties.  Yes  Very good  Waiting time is A LOT! It takes 2 hours or more  We pay on our own. |
| Conclusion   1. Is there anything you feel could be done to improve healthcare for your child or improve your experience?      1. Would you like to add anything before we conclude if you feel it’s important to you and I may have missed out? | For me everything has been very smooth Alhamdulillah! So can’t think of much.  No  Thank you!! |
|  |  |
