# Supplementary material for: Experiences of caregivers and healthcare providers regarding health services for children with Down syndrome in Karachi; Pakistan
Source: PLOS Glob Public Health. 2026 Apr 30;6(4):e0006225. doi: 10.1371/journal.pgph.0006225 (PMC13132430; doi:10.1371/journal.pgph.0006225)
Supplement: S1 Data — (ZIP) [file pgph.0006225.s001.zip › Minimal Anonymized Data transcripts/caregivers-CG1- For PLOS.docx]

After introducing the Principal Investigator (PI) and the research topic to the participant, the consent form was explained in detail. Written consent was then obtained from the participant for both participation in the research and audio recording.

**Participant ID: IDI-CG-XX**

**Date: 16^th^ Aug 2023**

| Can you give me a few details about yourself like name, age, education, marital status, age of your child with ds and socio-economic status (optional)? | My name is Dr.XYZ. I'm an assistant professor in anatomy, XYZ Medical College I'm in PFA base here.  I'm married. I have one daughter. (Name of Child). She is **4.5 years old**, she has Down syndrome. I am **33 years old.**  Alhamdulillah, we are well. Not very well, but very well. Alhamdulillah, Alhamdulillah. |
| --- | --- |
| 1. What is your relationship with the child?  - In case of mother/ father probe about the total number of children - In-case of sibling probe about the total number of siblings - Do you care for the child alone or do you have help? | ( the participant was the mother since she already covered that in the beginning of the interview there was no need)  One.  She is the youngest  No, her father is also there. This is my main role. |
| 1. Can you tell me at what age was your child diagnosed with Down syndrome?  - Who made the diagnosis? Was it in a clinic or hospital? - Were you aware of what it was? - Did your doctor counsel you about what it was and how to deal with him? - probe about possible questions that may have remained un-answered  1. What was the emotional impact of this news on you and your family? | She was diagnosed just after birth on clinical basis. And then on the second day of her birth, her karyotyping was sent. And after one month, it was positive with trisomy 21, 100%.  Lahore. She was delivered in Lahore. Within one month. Confirmed. My elder brother is actually a pediatrician. He received (name of child). I got emergency cesarean section. He received (Name of Child) in OT and he saw her features. On the basis of her features, he decided to send karyotyping on the very next day. And after one month, the karyotyping was trisomy 21.  No counseling from no one.  **(Did anybody tell you how to deal with this?)**  No one. Just my elder brother told me that she has low tone, you have to massage her. And there are therapies, but he told us what she had after two years of age. Then I got to know from YouTube sabout NGO. And we came here when she was four months old. And then we were Suddenly excited that she will do therapies from now. We were thinking that after 2 years she will be getting therapies. And till then I was doing her massages in home and some activities like from the applications, I downloaded applications how to activate your child. So I was playing with her whatever knew but they were for normal kids and not special kids like tummy time. No one referred us to NGO. We just saw the videos of the YouTube, what will happen, how the children are. Then from the YouTube we got to know that there was a center. I forgot that name, the special needs center. I contacted them, no response from the call. Then I contacted on Facebook, they replied me, the center has been closed and we are abroad now.  **was it a public center or a private center**? It was a private center. I forgot that name and we saw that video from the YouTube. They were in some like morning show. Morning show video, (**wahan se hamne dekha tha**). And then they referred to me the NGO name and we saw the videos of the NGO, we searched for the NGO and then we saw (name of owner).  I called many times (at the NGO), no one received my call. Then I messaged (name of owner) on Facebook and he replied me on Facebook that he was out of country those days. And then I got call from the NGO and then we came she was four months of age. And on the next week her physiotherapy was started.  At that time I was little emotionally weak I wept a lot with my husband and with my family, but there was no negative word from my tongue to Allah. That I promise I will not use any negative word why me or no, no these words, but I got to denial phase. Maybe her report has been changed, maybe sample, because report was, the Blood sample was, we took that report to the chugtai lab and they confirmed it from(name of private hospital) , Karachi. So, that was all, I think the sample was exchanged from Lahore to Karachi. I usually look my baby and her features in night, mostly. I used to pick it up and see the up slanting, the medical features, the nasal bridge, fingers, and all the examination I do at night and then I say, no, no, this is not right that’s , this is wrong. And sometimes when I used to see up slanting, I used to say, no, this is right. I mean, why am I denying myself? This is right. This is how it is. And she used to cry very little, she used to sleep a lot. So I used to think that, because other kids don’t do this. So I used to think that this is right. Initially it was a denial phase, then came the anger phase. I usually had many fights with my husband. And I used to think that maybe my husband left me due to the abnormal child. Maybe he thinks that I cannot deliver a normal child. Maybe he will go for a second marriage. All these thoughts were in my mind. So, I used to start a conversation with him and I used to be loud and aggressive. And then at the end I used to say that I will never leave this child, I can leave everything else. Family, if someone talks ill of my child, I will stop meeting them. It took me almost 6 months to recover. My husband used to counsel me, why would I leave you, I am with you, and she is my child, why would I leave her? I will not leave her. We will work on her We will get her therapies. We will make her independent. Because there was a child in our in-laws’ house. He was almost 10 years old. Everyone treated him very badly and abnormally. Because he did not take any therapy. He stayed at home. They neglected him a lot. He also had Down syndrome. When we were diagnosed with Down syndrome, we were only thinking about that child No one guided us. They have many children. They were not focused on their child. It was our first baby, so it was our main focus. When we came to NGO we got such good counseling here. They guided us so well. Then we started to see the ray of hope. So we first went to a physiotherapist. And on the first day, when we did the assessment, the therapist said that we can see that the baby is worked on at home. Because she has neck holding at that time. So, at six months she had sitting. So, she encouraged me a lot to continue therapy at home. She used to tell me to do 10 times, but I used to do 20 times and bring her. I mean, I did a lot of work in the beginning. When she started walking, it felt like a blessing. Earlier it felt like a struggle, now it feels like a blessing. When she responds, smiles, hugs, now it feels like a blessing |
| 1. How often do you visit hospital/ clinics for your child?  - probe about the distance and accessibility - Probe about any specific facility the caregiver visits and does someone accompany him/her? - Do you feel like your child is treated as any other patient during these visits? | My brother advised me to keep my child on breast feed. Minimum bottle feed. Because infections and all these are more in this. Immunity is less. So, (name of child) was on total mother feed. So, for two years, (name of child) did not have any infection as such which didn’t require for her to get admitted. For small problems, we had to go to the hospital. She had otitis media. We had to go for antibiotics. This continued for a month. Other than that, she had a slight fever due to teething. We haven’t even nebulized her.  So, her hospital visits are very limited.  After starting pre-school at NGO and interaction with other kids at the age of 2.5 years she has been exposed to multiple infections. She has been admitted twice with pneumonia, once with diarrhea and now with typhoid. Recently, last month. Over all four hospitalizations from that time and one hospitalization this year.  We go to (name of local hospital) which is near our home. My husband works in a panel company, in a Korean company. So, we have an insurance panel.  Yes, we both live in the hospital. They give us a room and we stay there.    No. (name of local hospital) people treat me very well. They are very friendly with (name of child). Whoever comes in, they say hello to( name of child), say hi to him, and shake hands with her. Most of them kiss her hand and leave. Doctors and paramedical staff. |
| 1. Does your child suffer from any specific health conditions which require for you to see a specialist periodically? (Thyroids, vision/hearing, cardiac related problems, mental health issues, delayed speech etc.?)   Only if the answers to the above question is yes:  Inquire about the type/ name of institute he is visiting and an idea of what kind of services are common for him to on those visits | No, we don't have any congenital problems. Like heart problems, or ear problems, or hearing problems. We didn't have any of these. The deafness was normal. GIT was normal. The barium was also normal. The echo was also normal. It was no issue. Alhamdulillah.  Since I am a doctor, I manage on my own however when she stops eating I have to approach a pediatrician (name of Doctor). They inject her with IVS but till the time comes I manage everything at home.  We visit a secondary care hospital with 100 plus beds with reasonable services. |
| 1. What challenges did you come across while trying to access healthcare for your child in Karachi?  - probe about structural difficulties in facilities( wheel chairs, elevators or anything that may be of use but wasn’t there) - What are the factors that made getting access to healthcare easier? - Do you feel like your child is getting all the relevant health services he/she may require - probe about the doctors behavior and support(?(covered in the statement in the beginning) - probe about waiting time ( answered in the beginning)  1. How do you manage to keep up with the financial aspect of getting health services for your child?  - Probe about support money from any organization or otherwise? | In the beginning, we used to go to(name of private hospital. It was on payment, but the charges were very high. The OPD service costed us around Rs. 3000. My brother told me that I had to get his eye checkup, ear checkup done yearly. And we had to visit a medical specialist and get her TSH, CBC done. We had to get all these things done yearly for 3 years. So we did all that but when we used to go to the private hospital, There was so much waiting, almost more than 30 minutes, 1 hour. And after that, only 5 minutes of consultation. The doctor gave us only 5 minutes. Then we felt that we had wasted 3000. they used to say “shes okay, why did you bring her” like We went to the ENT he said “shes okay, why did you bring her?”. He didn't use any instrument for the ear, nose, did not check for adenoids. He just asked me if there was a problem. We went for 3 years and then left. For 3 years we did eye checkups and ears from private hospital. We were told to get a Bara test. We got it done with Friends Optics. We got TSH and CBC done annually for 3 years. Now we have not done TSH, CBC, is done as soon as she is admitted.  I think so. Another thing is that people from (name of private hospital) know about Down Syndrome. They know. But wherever we go, the majority doesn’t know about the issues of Down syndrome. Like for example, (name of child)had a constipation issue. It was so serious that she went under barium enema. But it was not getting resolved. I went to the surgery people here and there and the last time I went to Dr (name of Doctor), he asked me about her water intake. I told him that that she does not drink water at all because she does not know how to swallow water. She would gag or he would have a reflux and would go into a breathless state. She was not able to handle thin liquids. That's why she didn't drink water. If we gave it, she would throw it out her mouth. Because of the low water content, she had constipation. I didn't understand that. I added everything to her diet. Vegetables, wheat, everything. I made smoothies and boil vegetables and mix them. Because my mother used to say that these two are good for constipation.  But, there are no such setups here to deal with our children's problems. I mean, when we go to other doctors, they don't know what problems they may have. Like, constipation is so common, ear infections are so common. But they take these things very lightly. They don't understand this because their immunity is less. The specialists like ENT, all year round have visits from streamline normal kids so they are unfamiliar with our children’s issue. I mean, there must be clinics, which will soley deal with kids like ours with professionals that understand their problems soley. So that they know that these children come for a reason. Like, all for all the families here there has to be a platform when it comes to health care. We know that we have to go to that set up for our child. For her ENT, for their eye. If they have any heart problem, we have to go there. There must be healthcare individuals that deal with them specifically.  No, they have to get it. On payment, nothing else. Yes, and that too, like my brother guided me, or NGO guided us. Parents who don't know, they don't have any services for their children.  In foreign countries, if a child is born with special needs, there are counseling sessions for the parents also. Here, there is no counseling. We know what phases we are going through. And after that, the society pressure that we are going through, it doesn't work. “Doesn’t she walk? a 10 month old child should walk!”. This child is 1.5 years old and still doesn't walk” “she doesn't speak?A 3-year-old child talks so much!”. And a 4 year old child doesn't speak??. You are not doing anything for her? “yes she will throw toys its natural for her”. Like in our society there is lack of awareness. Instead of showing empathy, they say such things. I say, O Allah, don’t put them through this test. Because if they go through it, we will know what stage we are in.  We have insurance (from my husband’s company) that covers hospital visits (opds), medication and some therapies not all. However there is a limit for the congenital. We have a limit for 40 thousand. We always exceed 20-30 thousand each year because of the therapies. We have to pay that out of pocket. Because her therapies cost 7 thousand a month. |
| Conclusion   1. Is there anything you feel that could be done to improve the healthcare for your child or improve your experience?        1. Would you like to add anything before we conclude if you feel it’s important to you and I may have missed it out? | I think that the way NGO is providing therapies and the financial assistance is very good. Hopefully in the future there are hospitals made for individuals with special needs, where all the services are available. And the ones who can afford it should take on payment and the others if not for free are offered services at subsidized rates. however such a project will only be funded  By( names of two welfare organizations or another NGO. The government won’t make one; even if it does there will be nothing in it.  We have never taken (Name of child) Ruhab to a government hospital. We went to (name of a renowned hospital) once (ojha campus) which is a semi-government campus for speech therapy The waiting was so long, and the speech therapist only gave us five minutes. We never went after that. We thought we would pay more but never come back.  We can afford treatment that’s why We did the assessments and all the tests. We had awareness. But for the non-affording parents It is very difficult for them to take care of a special need child. They have to keep him healthy. Because they don't have any services they are neglected and their milestones are delayed. After that, they go to small clinics or medical stores to get medicines. . The medical store people act as pseudo doctors. They give medicines and say, give him this, give him that. So maybe that's why their children are allergic. I have seen it myself. They cannot afford those services. They are neglected. They have the same delay. I think this is the matter. The government will not do anything. But those who can should do something. Thank you so much.  It was great talking to you. I learned a lot. Thank you so much |
|  |  |
