# Supplementary material for: Experiences of caregivers and healthcare providers regarding health services for children with Down syndrome in Karachi; Pakistan
Source: PLOS Glob Public Health. 2026 Apr 30;6(4):e0006225. doi: 10.1371/journal.pgph.0006225 (PMC13132430; doi:10.1371/journal.pgph.0006225)
Supplement: S1 Data — (ZIP) [file pgph.0006225.s001.zip › Minimal Anonymized Data transcripts/Healthcare provider PH1-for PLOS.docx]

After introducing the Principal Investigator (PI) and the research topic to the participant, the consent form was explained in detail. Written consent was then obtained from the healthcare provider for both participation in the research and audio recording.

**Participant ID: IDI-HCP-PH**

**Date: 30^th^ Aug 2023**

| Can you please tell me a few basic details about yourself: name, age, profession, years of experience etc.? | Alright, so my name is Dr. XYZ. I am 35 years old and working as a consultant pediatric in local healthcare facility and NGO as well.  I have 10 years of work experience. I am working as a consultant in XYZ Foundation and as a part-timer in NGO. So XYZ Foundation is a public-private partnership having multiple emergency units in town. |
| --- | --- |
| 1. How many children with DS do you have under your care? Or how often do you deal with a child having DS?   - Who accompanies these children mostly? - What is the age range in which diagnosis is commonly made and are any additional screening methods used for this purpose? - How would you define the level of awareness of parents/family about the condition of their child at your first visit? | I have been doing OPDs bi-monthly and in every OPD I have almost 5-6 patients. That makes 12 patients a month and almost 150 patients a year. So these are almost 30% patients that are on follow-up and 70% new patients. So If I talk about the NGO only I believe it is between 100 and 150.  Mostly by mother and father. Usually both of them are present.  Usually the initial diagnosis is made in first year of life. Mostly they are diagnosed in neonatal period when the baby is born. So I am finding that most of them have been diagnosed by the age of one year (0-12 months). So the basic test is karyotyping. In addition, we do screening tests for different systems like for heart and for eyes and for neurological status. So for diagnosing, there is one karyotyping that we are doing and for assessment, we have multiple assessments of different systems.  So basically we are lacking a lot of antenatal scans and antenatal visits for sure. And whoever is doing the antenatal scan, they are not looking for these things. This is only being done in good hospitals like “ Tertiary hospital name and “another private hospital name” however not at other primary or secondary hospital level neither in clinics.  So those patients who are coming at the first visit, so they don't know much about Down syndrome. So they have just a structure canvas that the baby is having low IQ, that is their main knowledge and they are living the most of the part that they like. Usually they come with very little knowledge about Down syndrome. I believe there are almost 60-70% patients who have less knowledge and almost 20-30% patients who are well knowledgeable. So more educated patients come with having knowledge. They go through Google and YouTube. |
| 1. Is there any statistical data on the prevalence/incidence of Children with Down syndrome in Pakistan that you are aware of? Who should be in charge of supplying such information (medical professionals, institutions, or other sources)?  2. Are there any guidelines for the initial medical counseling for parents of CWD at birth and/or at first exposure to the diagnosis, including prenatal counseling? Who is responsible for referring families to genetic-medical counseling for DS?  3. Which reference sources/guidelines are available/used in modern Pakistani healthcare practices?  Challenges with providing care  5. What makes it really difficult for you in providing care to these children and what makes it easier?  • Are your views easily communicated to the caregivers?  • How often do caregivers bring their child back for a follow up?  • What are the caregiver’s reactions on being referred to a different specialist in case the need arises?  6. Are you satisfied by the standard of healthcare and the services being offered to children with Down syndrome in Karachi, Pakistan  • Do you think something can be done for improving access to the relevant services?- Hr, HMIS or infrastructure  7. In your opinion what factors can make your job easier in providing health services to the children with DS?  • structural, individual , organizational or otherwise | So the data we are finding out is coming from Western countries, so we don't have the data from Pakistan as such. We need to have this data but I am not aware of any such data that is published in Pakistan. So we are following the data which is published internationally.  I don’t know any such guidelines.  The primary pediatrician should be the person who is sending the patient for genetic testing or follow up genetic testing.  Yes, there are different guidelines. We follow the European guidelines. So they ask us to screen different systems like heart and chest and eyes and ears at certain level, then we follow. So we really follow these European guidelines.  What makes it difficult is Firstly the availability of services or facilities and secondly determining whether the parents can afford it or not because mostly parents can’t afford the facilities and since they pay out of their own pocket they skip on ECHO, thyroid testing or even therapies. Affordability makes it very difficult.  What makes it easier is if we have some centers like NGO that provide good facilities and if someone cannot afford the services they provide it for some less cost or fee. So it would be good that if we have more platforms like it.  yeah, I believe it because they are coming for a general pediatric learning. They came with an open mind. They want to listen to what the pediatrician wants to tell them. So, yes, they follow the idea.  I advise them to come every six months otherwise bi monthly. so follow up is good.I believe they are following; we are referring patients for cardiac assessment and neurological assessment. So, they are already following a lot of Down syndrome patients. So, and they are quite senior to me. So, they are quite accepting and they know the challenges and they counsel them well.  So if I say that for example if a patient I am seeing here at Down Syndrome and a patient I am seeing in a public sector hospital like XYZ Foundation, so the parents who are coming here, they have more sense of responsibility and they understand things better than those who are coming in public sector hospitals. So, the parents who are coming here, they usually do follow this guideline. But those who are coming in public sector hospitals, because they don't have such facilities, they try to skip or dodge these follow-ups and advices.  No, I believe that there are certain things which we are improving like cardiac assessment and cardiac health. We have multiple units where we are assessing these patients well. But I believe that the rehabilitation part is quite low.  I am not actually satisfied but we are quite improving in some part of assessment like cardiac assessment we are improving in general but the rest of the rehabilitation phase is quite low.  I think we can take the help of social media because social media is quite vibrant. So we can spread the knowledge of social media by reaching these kids and then bringing them to centers where they can provide better facilities.  we have the facilities, we have the services, health services, but we just don't have the resources.  It is not utilized. It is not 100% utilized. So if these parents know that there is somewhere they can go and they can find all the things under one roof, I think it would make things easier for them and us.  “So I think the constellation of different sub-specialties under one group, that can help because the pediatrician has a very important but a minimum role. The major role is played by physiotherapists, occupational and occupational therapists and speech therapist. So pediatricians are the fulcrum between them but pediatricians like to have specialties around them which they can send easily. So, we should have liaisons with these subspecialties where we can send them. So that might help in improving their care”  There needs to be a network between pediatricians, an endocrinologist, a cardiologist a therapist. There needs to be a system. If not under one roof, then a system in place where we can make easy referrals.  We need to find ways to reach parents, we need to find ways to reach them so we can help their children with the relevant facilities |
