# Supplementary material for: Experiences of caregivers and healthcare providers regarding health services for children with Down syndrome in Karachi; Pakistan
Source: PLOS Glob Public Health. 2026 Apr 30;6(4):e0006225. doi: 10.1371/journal.pgph.0006225 (PMC13132430; doi:10.1371/journal.pgph.0006225)
Supplement: S1 Data — (ZIP) [file pgph.0006225.s001.zip › Minimal Anonymized Data transcripts/Healthcare provider-PH2-For PLOS.docx]

After introducing the Principal Investigator (PI) and the research topic to the participant, the consent form was explained in detail. Written consent was then obtained from the participant for both participation in the research and audio recording.

**Participant ID: IDI-HCP-PH**

**Date: 20^th^ Aug 2023**

| Can you please tell me a few basic details about yourself: name, age, profession, years of experience etc.? | My name is **Dr. XYZ** I am a **Neonologist. I** am also the section head for Neonatal Services at Tertiary care hospital; I am an assistant professor. I have about 20 years of experience. I am like a young age. I feel young at heart, so age is just a number. (participant is 40 plus) |
| --- | --- |
| 1. How many children with DS do you have under your care? Or how often do you deal with a child having DS?   - Who accompanies these children mostly? - What is the age range in which diagnosis is commonly made and are any additional screening methods used for this purpose? - How would you define the level of awareness of parents/family about the condition of their child at your first visit? - Do they belong to a specific socio-economic class?   1.Is there any statistical data on the prevalence/incidence of Children with Down syndrome in Pakistan that you are aware of? Who should be in charge of supplying such information (medical professionals, institutions, or other sources)  2.Are there any guidelines for the initial medical counseling for parents of CWD at birth and/or at first exposure to the diagnosis, including prenatal counseling? Who is responsible for referring families to genetic-medical counseling for DS?  3. Which reference sources/guidelines are available/used in modern Pakistani healthcare practices? ( not answered)  Challenges with providing care  5. What makes it really difficult for you in providing care to these children and what makes it easier?  • Are your views easily communicated to the caregivers?  • How often do caregivers bring their child back for a follow up?  • What are the caregiver’s reactions on being referred to a different specialist in case the need arises?  6. Are you satisfied by the standard of healthcare and the services being offered to children with Down syndrome in Karachi, Pakista  • Do you think something can be done for improving access to the relevant services?-  7. In your opinion what factors can make your job easier in providing health services to the children with DS?  • structural, individual , organizational or otherwise | I have maybe 10 children that are actively following but we do see babies with Trisomy 21 quite frequently. Not all of them do come in regular follow-ups with us.  By their parents mostly.  Our families are well into this and mostly both parents show up  So antenatal ultrasound has become quite sensitive in picking up features that are very suggestive of Down syndrome. So the first trimester ultrasound if you look at the nasal bone, the knuckle translucency and there are a couple of other features that are very highly suggestive of an aneuploidy. Aneuploidy is like any problem in the chromosome. But that alone does not substitute for an invasive or non-invasive testing. So, they are now recommended, like the ultrasound is a recommended thing to do, but even after the ultrasound, you have to go for an invasive or non-invasive test to diagnose in utero. So, we can actually diagnose Down syndrome in utero.  So there are two cohorts that we see. One is the ones that know that their baby has a problem and this can be a down syndrome and the other ones are caught off by surprise. So, they don't know and this baby delivers and we find out. These two families have a different approach. First family is more mentally prepared and because they knew that baby has a Down syndrome, so they chose to continue the pregnancy and once they choose to continue the pregnancy then they read about it and all. So it becomes a little easier for me to like for us to counsel them about the condition and all and they start off at a different level in terms of in terms of their journey to know how to take care of a trisomy 21 baby.  There are two types of families the ones that don't believe in abortion at all and they think that abortion is like killing a person so they continue irrespective of their financial, social, economic or religious beliefs they want to continue the other ones that mostly continue are the ones who don't have children Or have just only like maybe one child or so and they feel that, you know, I mean, something is better than nothing. And they think that, you know, and their awareness, because now Down syndrome, so they lead quite a normal life. So, I mean, so they know that, you know, even with Down syndrome, their child is going to be pretty okay. So they want to continue. So these are the two types of people I feel I have seen them to continue. The ones that want to continue, the first category I told you about, they don't have any social or demographic or education concept, they just have a religious belief, and that is what they do. The second category of individuals most of them from educated backgrounds who are unable to conceive and you know, been married for a long time, they don't have children, but when they conceive, they find out that the baby has Down syndrome and they make this decision to continue based on what they read and what they talk with people and all.  So the non-antenatally diagnosed, they find it very difficult initially to digest that there is something wrong or maybe not wrong, something different with their child.  Since my experience is only with the neonates (new born) and those antenatally diagnosed I can’t give you a range.  No, there is no registry here. There is no official registry. None on the government level. I think NGO is collecting some data.  In the West, mostly it is government-led organizations. But I am not sure that any private organization. There should be a registry, it can be led by anyone, like I mean it can be a government organization that looks after Down Syndrome specific, it can be a government private partnership, it can be an institution like us, who can you know set up a registry and people start reporting, it doesn't really matter but what matters is that we should have a registry and people should know about it and then every baby with Down's syndrome should get enrolled.  I follow my own pattern. There is no guideline. There are no guidelines. We follow based on what needs to be conveyed to the family. Every family is a little different.  Interesting question. There is no such ease in it. When you are breaking a diagnosis, it does not come across as very easy when we discuss with the family. So, it is not that easy. But, since we see a lot of kids, it is easy to talk and diagnose. The difficulty is that there are a lot of taboos in our society, gender-based taboos. Families think that, you know, women think that they might have done something wrong, because of which this happened. Husband thinks that wife did something; wife thinks that it was because of a solar or lunar eclipse so because of that down syndrome has occurred. There are many ridiculous myths.  The difficulty is that sometimes it is hard for them to digest and to acknowledge the fact that there's something different with their child, then the grief phases, the first phase is denial, they deny that it can't happen. Then comes the projection of the second phase, that this happened because of this, that because of that, that he did this, that he did that. The third phase is acceptance. So, acceptance sometimes needs time.  No, they are communicated but they are not accepted.  Sometimes.  they come back  Whenever they need to be.  Absolutely, so yeah. So, I thought maybe you were asking about the faculty. So no, so parents do find it difficult because first thing is you tell them the diagnosis. After the diagnosis you say that there are some things that can be there. So go to a cardiologist, go to a hearing test, go to an eye exam. So sometimes there is a little apprehension in that. But if there is a problem, there is more apprehension. If there is no problem, and screening is going on, then it is fine.  No, not at all. No, we do not have, I am not very satisfied. I think there is a lot to do and I think there is a lot of area of need, need to create awareness and need for people to advocate, need for resource allocation.  When we do screening in the first trimester, and we find out that the patient is at high risk, we offer invasive testing. Invasive testing and non-invasive. If something is detected in the testing, they go to genetics, they come to us, and stuff like that.  Honestly NGO is the only organization we refer them to and we get feedback about the children from them. They have a team so things are easily channelized.  I think by having a dedicated service, a one window solution in our hospitals where a child with down syndrome is immediately referred to it and his entire treatment plan is aligned and tracked. A special clinic especially for them. There used to be a clinic at aku previously where the entire DS team used to visit it in one sitting. I don’t think it’s functional or its turnover either ways I’m actually not sure.  There needs to be a package where services are free of cost, that’s not the case at aku but at least they should be state sponsored. I personally wish its everywhere a one window solution, very subsidized and free of cost and a registry so we can retrieve them and reach out to them otherwise patients will be lost. |
