# Supplementary material for: Experiences of caregivers and healthcare providers regarding health services for children with Down syndrome in Karachi; Pakistan
Source: PLOS Glob Public Health. 2026 Apr 30;6(4):e0006225. doi: 10.1371/journal.pgph.0006225 (PMC13132430; doi:10.1371/journal.pgph.0006225)
Supplement: S1 Data — (ZIP) [file pgph.0006225.s001.zip › Minimal Anonymized Data transcripts/Healthcare Provider-TH1-For PLOS.docx]

After introducing the Principal Investigator (PI) and the research topic to the participant, the consent form was explained in detail. Written consent was then obtained from the healthcare provider for both participation in the research and audio recording.

**Participant ID: IDI-HCP-TH**

**Date: 28^th^ Aug 2023**

| Can you please tell me a few basic details about yourself: name, age, profession, years of experience etc.? | My name is XYZ. I am a physiotherapist**.** I have experience of around 10 years in this field of pediatric physical therapy. And I have been working in the tertiary care hospital from 2012 to 2020. I resigned from the tertiary care hospital in 2020 during Covid season. And then I started my own private clinic. And after that in February 2023 I joined here in the NGO  Age: 33 |
| --- | --- |
| 1. How many children with DS do you have under your care? Or how often do you deal with a child having DS?   - Who accompanies these children mostly? - What is the age range in which diagnosis is commonly made and are any additional screening methods used for this purpose? - How would you define the level of awareness of parents/family about the condition of their child at your first visit? | I work full time at NGO. So I have in total 40 clients assigned to me on a weekly or bi-monthly basis. According to their condition at the level of their age. If they are good kids we are calling them twice a month that is bimonthly otherwise if they are little children/ younger children who still need work we call them on a weekly basis for guidance.  Mostly it's the mother.  Occasionally they are fathers as well    So mostly they are diagnosed from really early ages like within months.  This has happened with some of the families we are dealing with. They told us that they were diagnosed after some complication arose with their child. There were some developmental delays associated to the child Therefore they brought them to a doctor who then referred them to a specialist.  There have been very occasional few cases when parents give us history. That they have been diagnosed during ultrasound prenatally or any like after the birth. So children are diagnosed.  I have some cases that the parents went for NT testing. I think NT scan is done. There is an NT test and blood work is done So in blood work there is also DNA testing for Trisomy 21, 18 and different conditions like Edward syndrome is done. And then there is a growth scan. They can check your neck folds, your webbing, and your stature. Sometimes they can tell that the child has down syndrome. So there are multiple ways that the diagnosis can be done. You can even diagnose in utero.    There are children here who were diagnosed within 5 years, some in 3 years and most of them in the first year of birth |
| 1. Is there any statistical data on the prevalence/incidence of Children with Down syndrome in Pakistan that you are aware of? Who should be in charge of supplying such information (medical professionals, institutions, or other sources)?  2. Are there any guidelines for the initial medical counseling for parents of CWD at birth and/or at first exposure to the diagnosis, including prenatal counseling? Who is responsible for referring families to genetic-medical counseling for DS?  3. Which reference sources/guidelines are available/used in modern Pakistani healthcare practices?  Challenges with providing care  5. What makes it really difficult for you in providing care to these children and what makes it easier?  • Are your views easily communicated to the caregivers?  • How often do caregivers bring their child back for a follow up?  • What are the caregiver’s reactions on being referred to a different specialist in case the need arises?  6. Are you satisfied by the standard of healthcare and the services being offered to children with Down syndrome in Karachi, Pakista  • Do you think something can be done for improving access to the relevant services?- Hr, HMIS or infrastructure(the participant went off topic that part is omitted)  7. In your opinion what factors can make your job easier in providing health services to the children with DS?  • structural, individual , organizational or otherwise | In Pakistan. I don't know actually. I know that NGO is working on these things like prevalence and how many are there in Karachi. But I don't think that on the whole level like how many are there in Pakistan. I don't think so. Because when I came here, I refreshed my learning process. Obviously we learned about down syndrome in our undergraduate period. But when I came here, I did a refresher for myself. Before coming here, I knew that there are heart problems, GIT problems. So I refreshed my whole knowledge. But I didn't get any statistics here.  I think this information should be given by the hospitals in Pakistan. And the government should also have some statistical guidelines that they should give to every doctor “That you should see these things in the ultrasound the anomaly scan”  She means to say guidelines as to what to look exactly for in an ultrasound and an anomaly scan. As per my knowledge if the sonologist is experienced these anomalies can be determined through a normal ultrasound. Like web of the neck or the fingers. Webbing is a very good sign of Down syndrome because we do count the fingers and the toes during ultrasounds.  Yes, there are guidelines for counseling parents That show that children with Down syndrome have a very good chance for spending a very good quality life.    Whenever we search about Down syndrome, there are different guidelines. For example, there is America's APTA and NHS. So everyone has different guidelines. But it's not like they have made a specific guideline that you will only give this. For example, American Heart Association has different guidelines.  There are different guidelines for American Pediatric Association. There have been informative sources. But not specifically that you have to cover these things.  Usually after diagnosis we refer the child to a genetic counseller DR (name of female doctor) or DR (name of male doctor) mostly. The Diagnosing DR is a pediatrician or the primary physician must recommend the child to a genetic counselor especially if the child is very young because these children suffer from many co-morbid conditions (heart, brain and GIT) and we need to prepare parents as to what may happen in the future. This way these children are taken care of in a better manner. Counseling is very important in this regard no matter who does it. These children have very characteristic features and easily identifiable. I being a therapist can identify them which means a doctor should not lack. However we have had several cases here at NGO where the diagnosis is delayed for DS. The child went to the doctor for a heart condition and got diagnosed with DS at that time because of a comorbid.  What makes it difficult is that When parents come to us with preconceived ideas due to their socio-economic background or their cultural ideologies. These children lack knowledge and we have to guide the parents, we have to counsel them because as per my opinion there is this ideology that people tend to listen to only doctors more and not their therapists. What they don’t understand is the child sees us four times a month! I know the child better and not the doctor that sees him once a year or every six months.  What makes it easier is that being a part of the NGO has addressed so many of my issues which were not possible back in the tertiary care hospital (my previous organization) the organization facilitates these children so well. It would be easier for me if we worked as a team with doctors and different healthcare providers.  I have a very good follow up ratio. Out of 100 percent 90% do come back for a follow up. 10% fall out sometimes due to accessibility issues and affordability issues.  The usually get very scared. they ask questions like “ why” “what’s the need” and then a specialist consultation is so expensive, they are worried about how will they cover the cost”  There is a lot of room for improvement. If I talk about children on pediatric level things are already difficult so you can imagine how things are for children with Down syndrome. On a government level there is not much relief for children with DS even if there is something it’s not enough. Not everyone has dealt with such children, and since they reach their milestones like a normal child so people don’t realize it. Because of this many children come to us at the age of four years with speech delay problems because families weren’t guided and no one told them. The NGO reimburses parents with transportation costs if they can’t reach us but this is only for the 1500-1600 children with us, what about others? Where do they go?  When I was in aku and it has been an experience at my private practice where parents come to see me on the recommendation of some family or friends with the idea that physiotherapy will cure their child and that’s when I question them if they have ever been to a specialist and they say no, no one ever guided them and from there they were diagnosed with DS.  At the NGO we don’t have targeted therapy. We don’t focus on other issues that may be effecting the health of the child we don’t consider heart and other issues.  These children should be included. This needs to be addressed. The schools refuse to admit them which have an impact on parents’ mental health. It’s all about privilege.  Early intervention needs to be improved. There must be guidelines to detect metabolic and genetic syndromes. I know this is something in aga khan for high risk patients. There needs to be Genetic screening for high risk mothers in Pakistan not just for Down syndrome but for other conditions as well. In the first assessment by the primary doctor everything must be crossed out. Doctors are dismissive of the parents’ concerns saying “everything is okay everything is okay” the reality is nothing is okay. Parents on the other hand say “his father also walked when he was five years old” they keep delaying it because there are preconceived ideas and no guidance. we need to work on this and intervene as soon as possible |
