# Supplementary material for: Experiences of caregivers and healthcare providers regarding health services for children with Down syndrome in Karachi; Pakistan
Source: PLOS Glob Public Health. 2026 Apr 30;6(4):e0006225. doi: 10.1371/journal.pgph.0006225 (PMC13132430; doi:10.1371/journal.pgph.0006225)
Supplement: S1 Data — (ZIP) [file pgph.0006225.s001.zip › Minimal Anonymized Data transcripts/Healthcare Provider-TH2 for PLOS.docx]

After introducing the Principal Investigator (PI) and the research topic to the participant, the consent form was explained in detail. Written consent was then obtained from the participant for both participation in the research and audio recording.

**Participant ID: IDI-HCP-TH**

**Date: 18^th^ Aug 2023**

| Can you please tell me a few basic details about yourself: name, age, profession, years of experience etc.? | Okay, my name is XYZ, and I'm an *occupational therapist*, and I'm in the field of occupational therapy since last five years. And currently I am working in the NGO for the last eight to nine months. And my experiences start with the autistic children. The special children school schools, and then I joined (name of special school) special children school where my specialty is to conduct assessments and to provide therapy and to provide the treatment rules to the parents. And here I'm an occupational therapist. My age is 28 years old. |
| --- | --- |
| 1. How many children with DS do you have under your care? Or how often do you deal with a child having DS? 2. Who accompanies these children mostly? 3. What is the age range in which diagnosis is commonly made and are any additional screening methods used for this purpose?   4. How would you define the level of awareness of parents/family about the condition of their child at your first visit? | It's about 40 or 40 plus  We take therapies once a week, or some children who take therapies for a very long time and achieve their goals. So we offer them bimonthly mentoring sessions.  It's a mix up sometimes parents come sometimes Sisters sometimes Any guardian, like grandmother, uncles or anyone who is from the family. Mothers mostly.  When the child comes to us, he is already diagnosed however our team confirms that the child is in fact having down syndrome. Some children are pre diagnosed and some of them get diagnosed through us. We don’t have any testing here however we refer them to(name of Private Hospital); we have collaboration with( name of another local hospital) . The healthcare department can tell you better.  70-80% in fact 90% parents do not know what to do if they have a child with down syndrome in the family. We have a family awareness group working tirelessly to give awareness to the parents and guide them. We visit different hospitals to conduct surveys and ask them to refer a downs child to us.  Very few parents are aware of this, they get their information from the society “Haan woh ek idara hai jahan is tarhan keh bachon ko liya jata hai , is tarhan ka kaam karwaya jaraha hai”  Even When parents do visit us and we councel them, the parents the mother feels like the children are of no use to teach them is a waste of time and money. What we do is a waste of time these children cannot accomplish anything. so the parents are not very aware |
| 1. Is there any statistical data on the prevalence/incidence of Children with Down syndrome in Pakistan that you are aware of? Who should be in charge of supplying such information (medical professionals, institutions, or other sources) 2. Are there any guidelines for the initial medical counseling for parents of CWD at birth and/or at first exposure to the diagnosis, including prenatal counseling? Who is responsible for referring families to genetic-medical counseling for DS? 3. Which reference sources/guidelines are available/used in modern Pakistani healthcare practices? | As such no but the last research article I read stated that every 1 in 100 child is born with downs according to Pakistan’s population. But The population of downs has increased however the cause of it is still unknown.  I think there is a source I’m sure it’s just that I’m just not aware of it.  The healthcare and awareness department must be responsible for supplying this information. I think The research department from any hospitals or organizations should provide the healthcare providers or healthcare rehabilitators of this information. In occupational therapy We specifically counsel by our learning by our experience. There are no checklists or authentic guidelines, no statistics that we follow. We guide the parents as per our environment and society. All healthcare providers are responsible for referring the child to genetic counseling.  We use desi tools; we use sensory profile shortlist (not much but we are trying). These are not available in Pakistan; these are just standardized scales being used by us. We don’t have any guidelines of our own. |
| 1. **What makes it really difficult for you in providing care to these children and what makes it easier?**  - Are your views easily communicated to the caregivers? - How often do caregivers bring their child back for a follow up? - What are the caregiver’s reactions on being referred to a different specialist in case the need arises?  1. Are you satisfied by the standard of healthcare and the services being offered to children with Down syndrome in Karachi, Pakistan?  - Do you think something can be done for improving access to the relevant services?- Hr, HMIS or infrastructure(the participant went off topic that part is omitted) | It becomes difficult for us when we have children coming from a very low socio-economic background. To educate the parents, guide them, to tell them the pathway and how to follow it. These parents have very low morale because of the society. It becomes difficult for us as the parents are not accepting the condition and its requirements. It’s a slow process but we manage it eventually.  Yes  Mostly the follow up is good. Parents visit us weekly depending upon the time of their session. Similarly we give them guidelines and they also address all the problems they face in the follow-ups.  If we refer them to a neurologist, initially they don’t understand, and then we tell them “dimag ka doctor” The parents are taken aback and get suspicious of what has suddenly happened.  If we refer them to a behavioral therapist or a psychologist they think that the child is “mentally retarded”- the HCP used the words “koi pagal he jaye ga” This is where we have to counsel a lot to make them understand why the referral is needed and how the other healthcare provider can be beneficial for their child. Before sending them to a neurologist or cardiologist we need 2-3 days to prepare them.  No In Pakistan there is no other institute working with children having down syndrome apart from NGO in terms of various services inclusive of healthcare. In Rehabilitative departments other comorbid conditions are not referred continuously and do not provide quality services. If you remove the NGO there is nothing in Pakistan.  I think all rehabilitative centers should get the patients registered with the NGO so that this downs population can be on a single platform and something better can be done for them in every context. |
| 7.In your opinion what factors can make your job easier in providing health services to the children with DS? structural, individual , organizational or otherwise | We take 8 sessions in a day, if I’m allowed to take 5-6 sessions in a day it makes me more productive in terms of the quality of session I provide.  There are currently 1300 families registered with kdsp and 28 in-house therapists and two online ones.  Thank you so much! I learnt a lot. |
